# AGE INDUCES AN EMERGENCE FROM MELANOMA DORMANCY

**DOI:** 10.1093/geroni/igz038.2824

**Published:** 2019-11-08

**Authors:** Mitchell Fane

**Affiliations:** The Wistar Institute, Philadelphia, Pennsylvania, United States

## Abstract

Melanoma cell dormancy is regulated by intrinsic cues that maintain a slow-cycling state in cells and by extrinsic interactions with stromal and immune components of the microenvironment. A key factor is that a significant lapse of time occurs between initial diagnosis, and metastatic recurrence of dormant cells. During this time, the organism ages and age is a poor prognostic indicator for cancer. We have found that melanoma cells form lung metastases more efficiently in aged mice and remain dormant in young mice. Analysis of the immune-microenvironment of the lung reveals that healthy young and aged mice have little difference in infiltration of immune subpopulations; however aged tumor bearing mice have significantly increased immunosuppressive MDSCs and Tregs and decreased CD4+ and CD8+ t-cells in the lung compared with young mice. Our data indicates that aging induces an immunosuppressive lung microenvironment which allows immune-evasion and an emergence from melanoma dormancy.

